# HIV prevention cost-effectiveness: a systematic review

**DOI:** 10.1186/1471-2458-9-S1-S5

**Published:** 2009-11-18

**Authors:** Omar Galárraga, M Arantxa Colchero, Richard G Wamai, Stefano M Bertozzi

**Affiliations:** 1 Center for Evaluation Research and Surveys, Mexican School of Public Health/National Institute of Public Health (INSP), Av. Universidad 655, Cuernavaca, Mexico CP 62508; 2 Haas School of Business, University of California, Berkeley, CA, USA; 3 Department of African-American Studies, Northeastern University, Boston, MA, USA; Harvard School of Public Health, Cambridge, MA, USA; Nairobi University, Department of Community Health, Nairobi, Kenya; 4 Center for Economic Teaching and Research (CIDE), Mexico City, Mexico

## Abstract

**Background:**

After more than 25 years, public health programs have not been able to sufficiently reduce the number of new HIV infections. Over 7,000 people become infected with HIV every day. Lack of convincing evidence of cost-effectiveness (CE) may be one of the reasons why implementation of effective programs is not occurring at sufficient scale. This paper identifies, summarizes and critiques the CE literature related to HIV-prevention interventions in low- and middle-income countries during 2005-2008.

**Methods:**

Systematic identification of publications was conducted through several methods: electronic databases, internet search of international organizations and major funding/implementing agencies, and journal browsing. Inclusion criteria included: HIV prevention intervention, year for publication (2005-2008), setting (low- and middle-income countries), and CE estimation (empirical or modeling) using outcomes in terms of cost per HIV infection averted and/or cost per disability-adjusted life year (DALY) or quality-adjusted life year (QALY).

**Results:**

We found 21 distinct studies analyzing the CE of HIV-prevention interventions published in the past four years (2005-2008). Seventeen CE studies analyzed biomedical interventions; only a few dealt with behavioral and environmental/structural interventions. Sixteen studies focused on sub-Saharan Africa, and only a handful on Asia, Latin America and Eastern Europe. Many HIV-prevention interventions are very cost effective in absolute terms (using costs per DALY averted), and also in country-specific relative terms (in cost per DALY measured as percentage of GDP per capita).

**Conclusion:**

There are several types of interventions for which CE studies are still not available or insufficient, including surveillance, abstinence, school-based education, universal precautions, prevention for positives and most structural interventions. The sparse CE evidence available is not easily comparable; thus, not very useful for decision making. More than 25 years into the AIDS epidemic and billions of dollars of spending later, there is still much work to be done both on costs and effectiveness to adequately inform HIV prevention planning.

## Background

After more than 25 years into an epidemic of a completely preventable disease, public health programs have not been able to sufficiently reduce the number of new HIV infections around the world. The global AIDS epidemic update reports that 2.7 million people acquired HIV during 2007 [1]; over 7,000 people were infected with HIV every day [2]. Prevention may save millions of people, but it is far from working well. One of the reasons why implementation of effective programs is not occurring at sufficient scale is because of insufficient evidence of the cost-effectiveness (CE) of prevention interventions and programs [3].

Cost-effectiveness analysis (CEA) is a comparison of two or more alternatives in terms of their costs and effectiveness through a CE ratio: the difference in costs over the difference in effectiveness. Mathematical and epidemiological models are often required to simulate final outcomes from available data on intermediate outcomes. To make comparisons across different types of interventions and countries, CEA often uses a summary measure of effectiveness such as the quality- or disability-adjusted life years (QALYs/DALYs) gained/averted through interventions [4,5]. CEA can help decision-makers allocate resources and define priorities among a range of interventions.

Many studies on the CE of HIV-prevention interventions have appeared in the past four years (2005-2008), making substantial contributions to the literature since the publication of the *Disease Control Priorities Project *(DCPP) [3] and other reviews [6,7]. New interventions have emerged, increasing the debate for assessing resource allocation [8]. Furthermore, there are imperative reasons to conduct a new CE review. First, a dearth of CE data is available for HIV prevention [9,10]. Second, there is a need to make the limited, existing CE data available to program managers and implementers. Third, we need to update regularly the existing data to facilitate access: it may be difficult for decision-makers by themselves to easily absorb all the material that has appeared in recent years.

The objective of this paper is twofold: first it systematically reviews and summarizes the recent literature in terms of CE; second, it critiques and analyzes the comparability of the recent CE studies.

## Methods

The electronic databases utilized for the literature search were: Web of Science, Social Science Citation Index, PubMed (National Library of Medicine) and the American Economic Association's electronic bibliography of economic literature (EconLit). On-going projects by UNAIDS, World Health Organization (WHO), PEPFAR, and Global Fund to Fight AIDS, Tuberculosis and Malaria were also probed.

In addition, we reviewed the following scientific journals to find any articles not identified through keywords via electronic means: *AIDS, BMJ, Bulletin of the World Health Organization, Cost-effectiveness and Resource Allocation, Health Policy and Planning, International Journal of STDs and AIDS, JAMA, Journal of Acquired Immune Deficiency Syndrome, PLoS Medicine, Sexually Transmitted Diseases, and The Lancet. *Finally, suggestions from experts in the field complemented our search strategy.

Using the search terms and restrictions listed in Table 1 we retrieved 506 references. A preliminary analysis of abstracts was conducted, and led to the retrieval of 90 works for full-text assessment.

**Table 1:** Search Terms and Restrictions

Domain	*Description*	Search terms
Economic/evaluation	*Economic and Impact evaluation*	Cost, costing, effectiveness, cost-effectiveness, prevention, impact, HIV, AIDS
	
	*Setting*	Low- and middle-income countries (as per World Bank definition [11]); developing countries, third-world countries,, limited-resource settings (exclusion: high-income country)
	
Intervention	*Prevention interventions*	HIV/AIDS; school-based education; abstinence education; voluntary counseling and testing (VCT); peer-based programs; condom promotion and distribution; information, education and communication (IEC); condom social marketing (CSM); sexually transmitted infection (STI) treatment ("positive prevention"); antiretroviral treatment/therapy (ART); mother-to-child HIV transmission (MTCT) interventions; feeding substitution; harm reduction; needle exchange; drug substitution; blood safety; universal precautions; post-exposure prophylaxis; women empowerment; behavior-change programs; efficacy and effectiveness; structural interventions; social interventions; self-help and support groups; male circumcision (MC).
	
Publication Dates	*January 2005 to* *December 2008*	

We systematically reviewed the literature for CEA studies that compared a new intervention or program modification with a comparison case (no-intervention, status quo, or other intervention). The studies were classified by type of intervention (behavioral, biomedical or structural) as well as the specific prevention intervention. Based on these criteria, we reviewed 21 distinct CE studies.

The information about costs and effectiveness was abstracted into a checklist for each of the studies fulfilling the search criteria. We noted the place of the intervention, the target population, and the main issue addressed, as well as the type of epidemic in which the program took place (concentrated, generalized) and the main CE results. We reported CE studies that used cost per HIV infection averted (HIA) or cost per DALY/QALY averted/gained as the main outcome variable.

The classification of interventions in this review comes from a typology used by the UNAIDS and the World Health Organization (WHO) [9] and recent reviews on HIV prevention published in the *Lancet *[10,12-14]. Nevertheless, some of the interventions fall under two or more of the types presented. A better typology should recognize the independence of intervention and target population. The lack of clarity in intervention typology is most acute in the area of structural intervention, rendering it more difficult to speak of CE of different interventions. Although efforts are underway [15], more explicit definitions of what specific services come under each heading are needed to improve CE comparability across countries and programs.

High-income countries were excluded because of several reasons: the bulk of the new infections come from low- and middle-income economies, and thus it is in those countries where the largest impact in terms of HIV infections averted can be made; more developed countries have more resources to spend on health and with relatively less scarcity comes less competition for life-saving interventions; the differences in transmission types and the socio-cultural context warrant a separate analysis by income level [1,16,17].

To summarize the information obtained from the individual studies, we created graphs depicting the HIV-prevention interventions in terms of their cost per DALY [18], both in absolute terms and as percentage of the country-specific per-capita gross domestic product (GDP) [19].

## Results

Table 2 presents a summary of the studies addressing CE of the HIV-prevention interventions, by type of intervention. From the 21 distinct CE studies identified, five addressed behavior change interventions; seventeen dealt with biomedical interventions; and three analyzed structural/environmental interventions (the total number of interventions studied adds to 25 because one study dealt with several interventions at once).

**Table 2:** HIV Prevention Cost-effectiveness studies, 2005 - 2008

INTERVENTION	*# of studies*	REFERENCES
***Behavior Change***		

• Voluntary counseling and testing	*3*	Hausler, Sinanovic et al 2006; Hogan, Baltussen et al. 2005; John et al 2008 [20-22].
• Treatment for addictions	*1*	Vickerman, Kumaranayake et al. 2006 [23].
• School-based interventions	*1*	Hogan, Baltussen et al. 2005 [21].
***Biomedical Interventions***		
• Antiretroviral therapy	*1*	Over, Marseille et al. 2006 [24].
• Prevention of Mother-to-Child Transmission	*5*	Hogan, Baltussen et al. 2005; Reynolds, Janowitz et al. 2006; Soorapanth, Sansom et al. 2006; Maclean and Stringer 2005; Teerawattananon, Vos et al. 2005; [21,25-28].
• Treating STIs	*5*	Hogan, Baltussen et al. 2005; Vickerman, P., F. Terris-Prestholt, et al. 2006; Price, Stewart et al. 2006; Oster 2005; White, Orroth et al. 2008 [21,29-32]
• Male Circumcision	*5*	Kahn, Marseille and Auvert 2006; Gray, Li et al 2007; Martin, Bollinger et al. 2007a; Martin, Bollinger et al. 2007b; White et al 2008 [33-37].
• Female Condom	*1*	Dowdy, Sweat et al. 2006 [38,39].
***Structural/Environmental Interventions***		
• 100% Condom	*1*	Sweat, Kerrigan et al. 2006 [38,39].
• Women empowerment/Social/Peer-based programs/mass media	*2*	Hogan, Baltussen et al. 2005; Fung, Guinness et al. 2007 [21,40].

In terms of the regional and epidemic type (defined in Additional File 1), we found sixteen studies from sub-Saharan Africa (low- and high-level generalized epidemics), four from Asia (low-level generalized and concentrated epidemics), two from Latin America (concentrated epidemics) and only one from Eastern Europe (concentrated epidemic). The total is 23 because two studies dealt with two regions. See Additional File 1.

In the remainder of this section, we summarize the findings by type of intervention.

### Behavior change interventions

The aim of behavior change interventions is to reduce the risk of HIV infection through modification of sexual and addiction-related behaviors [14]. Studies were found in this category for voluntary counseling and testing (VCT), treatment for addictions (alcohol and drugs), and school-based programs.

#### Voluntary counseling and testing

A study in Cape Town, South Africa [20], measured the costs and CE of the ProTEST, a package aiming to decrease HIV transmission through voluntary counseling and testing (VCT). The authors collected annual cost data retrospectively using ingredient-based costing in three primary care facilities, and estimated the cost per HIV infection averted (HIA) and the cost per TB case prevented. The estimated cost per HIA by VCT in ProTEST was US$ 67 in the STI clinics and US$ 112 in the community clinics [20]. Although they found only moderate adherence, the link between prevention and care interventions for tuberculosis (TB) and HIV resulted in the estimated costs of preventing TB being less than previous estimates. VCT was found to be less expensive than previously reported elsewhere in Africa, primarily because the cost per client for post-test counseling was lower in STI clinics than in the other facilities because of the high number of people being tested and the use of lay counselors.

A generalized CE estimate for VCT in sub-Saharan Africa reported US$ 1,315 per HIV infection averted and US$ 82 per DALY [21]. Generalized CE refers to studies where the reference case is the null set, or no intervention; contrasting with intervention-mix constrained (IMC) CE, where the reference case is the current program or the status quo [41]. Some of the CE estimates have been estimated for regions as a whole, even though the effectiveness of the basic programs has not been conclusively demonstrated. The VCT intervention evaluated in the study was assumed to be performed in a primary care setting for any client who requested services. The costs included health-worker training, and rapid tests. The VCT impact was based on the number of individuals expected to complete the testing and the regional risk group-specific HIV prevalence.

A study from Kenya [22] found that infant HIV infections can be averted for less than US$ 483 per HIA using either of the two options explored: individual or couple counseling. The study reported that voluntary VCT for couples, although more expensive, averted more infant infections. The cost per DALY for couple VCT was similar to that of individual VCT. The sensitivity analyses found that couple VCT became more cost-effective as HIV prevalence and/or uptake of couple counseling increased.

#### Treatment for addictions

Vickerman and colleagues [23] explored the CE of a harm-reduction intervention among injecting drug users (IDUs) in Odessa, Ukraine, including the implications of successfully scaling up the intervention to reach 60% coverage as per WHO/UNAIDS guidelines. They used a dynamic mathematical model to fit the epidemiologic data and project the intervention's impact. Using the 1999-2000 coverage of 20-38% and an HIV prevalence among IDUs of 54%, 792 HIV infections could be theoretically averted, a 22% decrease in IDU HIV incidence. The cost per HIV infection averted would be US$ 97. Scaling up the intervention to reach 60% coverage would remain cost-effective and it would reduce HIV prevalence by 4% over a 5-year horizon [23].

#### School-based interventions

A generalized CE study determined the CE of school-based education (against the counterfactual of no intervention) as follows: US$ 6704-9448 per HIA (or 376-530 per DALY) in Africa; and 7288-13,326 per HIA (or 432-790 per DALY) in South East Asia [21]. The target population was school-age youth (10-18 years old); sessions were provided during regular lessons to all students with the aim of promoting prevention of HIV and other STIs; teacher-training costs were included. The variation in incremental cost-effectiveness ratios (ICERs) resulted from using different assumptions about coverage rates.

### Biomedical interventions

Biomedical interventions harness technology to prevent HIV/AIDS. These comprise chemical and physical strategies targeting biological and physiological processes that are responsible for HIV acquisition and transmission [13]. These include interventions already in widespread use as well as ones recently introduced and under development (e.g., male and female condoms, blood screening, treatment of STIs, pre- or post-exposure ART, PMTCT, male circumcision, microbicides, vaccines, and vaccination against viral infections, such as the human papillomavirus, herpes and hepatitis A and B).

#### Antiretroviral treatment and prevention of mother-to-child transmission

A generalized CE study reported the CE of prevention of mother-to-child transmission (PMTCT) to be US$ 34 per DALY averted in Africa and US$ 310 per DALY averted in Asia [21]. Another study analyzing antiretroviral therapy (ART) and prevention in India reported a CE range of US$145-280 per DALY averted with ART if enhanced with any of three prevention strategies considered: improved adherence to therapy; treatment for PMTCT+ (the + indicates that treatment would be available also for eligible husbands); and subsidies for ART for people living below the poverty line [24]. If antiretroviral treatment could be delivered in a way that also scaled up prevention efforts (mainly through increased condom use), the costs per life saved dropped to about US$ 10-30.

Because of practical and/or ethical concerns, actual interventions cannot always be implemented with all the ideal features for an evaluation. Modeling, then, becomes a useful alternative. One such case is a study that modeled prevention options in a hypothetical generalized high-level epidemic in sub-Saharan Africa, and estimated a cost of US$ 857 per HIV-positive birth averted with a single-dose nevirapine regime [25]. Interestingly, the study showed that prevention of unintended pregnancies would cost US$ 663 per HIV-positive birth averted; thus, family planning was a more cost effective way of preventing mother to child transmission than nevirapine-based HIV prevention.

A decision analysis model [26] carried out from the perspective of the health-care system in a generalized, high-level epidemic (such as South Africa and other resource-limited settings) assessed the CE of HIV rescreening during late pregnancy, using prevention of perinatal HIV transmission as the main outcome. HIV rescreening in the third trimester of pregnancy was found to be cost saving when ART was available for HIV-infected newborns.

Providing ART to mothers who are breastfeeding has also been analyzed from a CE perspective in the context of a hypothetical cohort of 40,000 pregnant women in sub-Saharan Africa. Providing daily infant NVP and breastfeeding for 6 months was the economically preferred strategy with an ICER of US$ 79 per QALY gained [27].

A study from Thailand [28] modeled a hypothetical cohort of 100,000 pregnancies and used a decision tree with various program options. The cost and outcome data were based on Thai settings. The combination of one and two VCT sessions, and four antiretroviral therapy regimens led to eight case options. One VCT session with AZT+NVP would avert 337 cases of infection at US$ 556 per case averted; thus, the program combining AZT and NVP had the lower cost per pediatric HIV infection averted.

#### Treatment of sexually transmitted infections

Controlling STIs has continued to be a policy option for HIV prevention around the world and a number of new studies have emerged. A study assessing an STI program for female sex workers (FSWs) in hotels in Johannesburg, South Africa, found the intervention effective in averting HIV infections [29]. The full intervention included condom distribution, treatment of symptomatic STIs and periodic presumptive treatment (PPT). A mathematical model fitted to the epidemiological data estimated that the cost per HIA was US$ 2,093 (range, US$ 1,384-3,635), or US$ 78 per DALY averted (with a range of $53-121).

A modeling study in Malawi involving a semi-urban population of men treated for *Trichomonas vaginalis*, where HIV prevalence was 44%, showed that treating all men would result in a decrease of 4.5% in the number of new HIV infections; trichomoniasis was responsible for 27.2 new cases of HIV in this sub-group population [30]. The costs of screening for trichomoniasis ranged between US$ 2 and US$ 62 per infection averted. The CE results showed that with 100% coverage the ICER over the status quo was $15.42 per HIA.

More generally, a modeling exercise for the African continent as a whole [31] reported that an intervention affecting HIV transmission rates through improved STI treatment could save 291 million life years with 13 million infections averted. That could be achieved at a cost of around US $78 per infection.

An epidemiological model simulating four HIV epidemics in West and East Africa [32] estimated the population-attributable fractions of incident HIV attributable to STIs. The cost per HIA range was US$ 321-1,665. The authors concluded that curable STI interventions remained cost-saving when compared to lifetime HIV treatment costs (of $3,500) in generalized HIV epidemics, in populations with high-risk behaviors or with low male-circumcision rates.

#### Male circumcision

Unlike other HIV prevention strategies, male circumcision (MC) is a one-time procedure with lifelong protective benefits and thus potentially highly cost-effective. The first CE study of MC used a mathematical model to simulate population-level MC in Gauteng, South Africa where one of the initial randomized, controlled trials (RCTs) was conducted [33]. Using a hypothetical group of 1,000 men 18 years and older, the study estimated the CE of providing MC services per HIV infection averted to be US$181. The cost of performing one MC (including medical costs of the procedure, community publicity as well as cost of treating adverse events) was estimated to be US$ 55. With a population prevalence of 25%, over a 20-year period, the 1,000 MCs would avert 308 adult HIV infections resulting in US$2.4 million in net savings due to the HIV medical costs averted over a lifetime. According to the study, the higher the MC coverage, the more cost-effective the intervention would be.

Another study conducted for a cohort in Rakai, Uganda used a stochastic model to simulate the impact of MC on HIV incidence, infections averted and cost [34]. The researchers developed different estimates for various levels of efficacy (40-60%) and circumcision coverage (25-100%). With the cost per HIA ranging between US$ 1,269 and US$ 3,911 at 75% coverage, the study found MC to be cost-effective even at a higher cost of US$69 per circumcision.

Studies by Martin, Bollinger and colleagues in Lesotho and Swaziland [35,37] provide MC cost and CE estimations. Both country-case studies used the same cost-estimation methodology modeled on an all-ingredients analysis of providing MC to 15-49 year old men at a coverage rate of 52.5% (Lesotho) and 57.5% (Swaziland) during the next twelve years (2008-2020). CE was assessed using cost per HIA and number of HIA per circumcision. The CE was found to be US$ 292 in Lesotho and US$ 176 in Swaziland. One HIV infection would be averted for every 6.1 and 4.1 circumcisions, respectively, in Lesotho and Swaziland. It is notable that the CE analysis was dependent on when in the roll-out the estimation was made, as well as the pace of implementation. The results showed that MC would be a highly cost-effective intervention.

An individual-based model fitted to the characteristics of an illustrative high-HIV-prevalence population in sub-Saharan Africa [36] found the costs (in 2007US$) per HIA in adults, by the intervention targeted at 15-49 year old men, over 2, 20 and 50 years to be US$ 1,806 (1327-3554), 195 (143-356) and 89 (71-150), respectively. Over the first 10 years of the intervention, the CE estimate was highest if the targeting was directed at 25-34 year-old men. Moreover, targeting any adult-age group was estimated to be a cost-saving strategy when compared to HIV lifetime treatment costs. In the short-run, however, targeting newborns or young males before their sexual debut was not cost-saving because circumcision occurred many years before men experience their highest HIV infection risk. Nevertheless, the intervention directed at neonates became as cost-effective as targeting adults after 20 years.

#### Female condom

Dowdy and colleagues [38], in a study of the CE of the second-generation female condom (nitrile female condom or FC2), showed the usefulness of analyzing the synergies between current unit costs and current CE vs. volume discounts or global purchasing arrangements and future CE. The results showed that in Brazil an HIV infected could be averted for US$ 20,683. In South Africa the ICER was US$ 985 per HIV infection averted.

### Structural interventions

Structural interventions attempt to change the underlying determinants of risk, vulnerability or disease. These programs may be called social, environmental, ecological, or upstream interventions. They are varied in nature and include changes in laws, prices and/or taxes, subsidies, vouchers, housing, income-generating activities, women empowerment, etc. [12].

#### 100% condom

Sweat and colleagues [39] analyzed how laws with strong consequences, and with positive rewards, can be instrumental in achieving better HIV prevention results in the female sex industry in the Dominican Republic. They found that the cost per HIA was US$ 10,856; or US$ 457 per DALY in Puerto Plata (structural approach with legal changes); versus US$ 28,208 per HIA and US$ 1,186 per DALY saved in Santo Domingo (traditional information, education, and communication, IEC) [39].

#### Empowerment/social/peer-based programs/mass media

Work by Fung and colleagues [40] reports on a prevention intervention for commercial sex workers (CSW) comparing changes in sexual behavior and condom use in Ahmedabad City (the seventh largest city in India) in which rates of HIV prevalence are particularly high among CSW. The CE study included four strategies with peer educators: increasing knowledge of HIV/AIDS and STIs, improving STI treatment of CSW and their clients, increasing safer practices, and environment improvement. All strategies were compared with no intervention. The costs per infection averted ranged from US$ 33.7 to $133.4 when peer educators where valued as financial costs, and from $55.6 to $128.5 when considered as economic costs. Similarly, the cost per DALY averted was: US$ 3.1 (1.9-7.5) to 5.5 (3.1-12.3) for the two scenarios.

A generalized CEA [21] of a mass-media campaign estimated the cost per HIA at US$ 58 per HIV infection or US$ 3 per DALY (ICER compared to no intervention). The campaign analyzed included television and radio episodes and inserts in the most influential newspapers, repeated every two years; development and administration costs were included; the effectiveness was scaled by proportion of population reporting weekly exposure to radio, television, or newspapers.

### Summarizing the HIV prevention cost-effectiveness results

**Figure 1:**
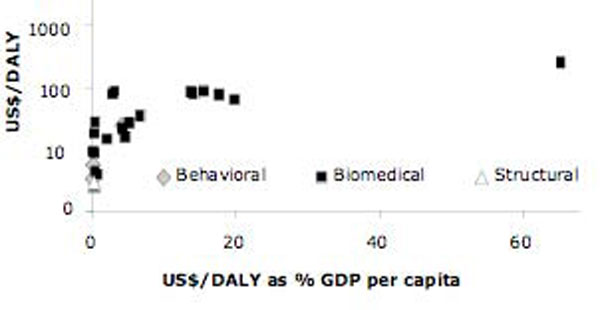
**HIV Prevention Cost per DALY vs. percent GDP per capita (Africa)**. Notes/Sources: The graph plots the studies reviewed in terms of US$ cost per DALY and the same US$/DALY as percent of country-specific per-capita gross domestic product (GDP) taken from the International Monetary Fund, World Economic Outlook Database [19]. US$/DALY is the cost per disability-adjusted life year, DALY (US dollars, year of costing as reported in each study, log transformed). When cost/DALY was not available in the studies, we assumed it was equal to the cost per infection averted/20 for adults, 25 for children [3,18]; one study used cost per QALY [25]. For details of each study, see Additional File 1.

Figure 1 shows a summary of the CE studies of HIV-prevention interventions in Africa, classified by type of intervention (behavioral, biomedical or structural). On the horizontal axis we have cost per DALY as the percentage of the country-specific per-capita gross domestic product (GDP) at current US dollars (of the year of the costing, as specified in each study) [19]. On the vertical axis we have the cost per DALY (log transformed). The figure shows that most of the studies of HIV prevention CE for Africa are of biomedical interventions. Some variability is observed, but generally the interventions are highly cost effective: most fall below a threshold of US$100/DALY. Similarly, the great majority of interventions cost less than 30% of one per-capita GDP and only one costs more than 60% of one per-capita GDP. Health interventions are deemed highly cost effective if they are below a threshold of one (or 100% of) per-capita GDP [42].

Moreover, Figure 1 shows a clustering of biomedical interventions for the African region. The clustering mostly represents a number of recent PMTCT and MC studies, which are clearly some of the most cost effective options among the different HIV-prevention interventions.

**Figure 2:**
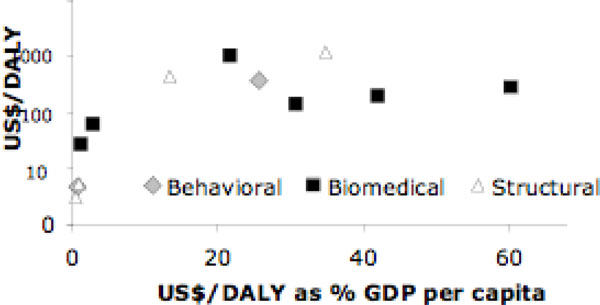
HIV Prevention Cost per DALY vs. percent GDP per capita (other regions*). Notes/Sources: *Other regions are Latin America, Asia and Central Europe (Ukraine). The graph plots the studies reviewed in terms of US$ cost per DALY and the same US$/DALY as percent of country-specific per-capita gross domestic product (GDP) taken from the International Monetary Fund, World Economic Outlook Database [19]. US$/DALY is the cost per disability-adjusted life year, DALY (US dollars, year of costing as reported in each study, log transformed). When cost/DALY was not available in the studies, we assumed it equals cost per infection averted/20 for adults, 25 for children [3,18]; one study used cost per life year [24]. For details of each study see Additional File 1.

Similarly, Figure 2 shows a summary of the HIV-prevention interventions in regions other than Africa (i.e., Latin America, Asia and Central Europe), classified by type of intervention (behavioral, biomedical or structural). The axes are the same as in Figure 1. Most of the studies of HIV prevention CE for other regions are also for biomedical interventions. Some variability is observed, but generally the interventions are cost effective: a few interventions cost about $1,000/DALY but most fall well below that threshold; and in relative terms, most cost less than 50% of one per-capita GDP, and only one intervention was about 60% of GDP per capita. Note that some of the interventions in other regions (in contrast to Africa) cost relatively more, when compared to their own GDP per capita; this is related to higher expenditures in health and the lower HIV prevalence rates. Nevertheless, the general distribution of the CE results is strikingly similar between Africa and other regions.

## Discussion

A prior HIV review presented in the book on *Disease Control Priorities in Developing Countries *[3] found no CE studies in the areas of: surveillance, IEC, MTCT feeding substitution, IDU drug substitution, universal precautions, vaccines and behavior change for HIV positive people. In this review, we have found newer CE studies which cover some of the previously unexplored areas: IEC [39], behavior change among people initiating ART [24], male circumcision [33-37] and harm reduction for IDUs [23]. However, there are still multiple interventions whose CE has not been assessed and several more for which the evidence is very limited. There continues to be a lack of any CE work on prevention for or with positives, which has become more important as HIV treatment expands throughout the world. Most of the studies reviewed focused on sub-Saharan Africa; only a handful were based in Asia and Latin America. As there are fewer studies for concentrated epidemic settings, it is not surprising that we found no CE studies for the most vulnerable groups in concentrated epidemics. For example, there is still no CE evidence regarding men who have sex with men (MSM) or male sex workers (MSW) in Latin America, or any studies about inmates or other captive populations.

An examination of the summary graphs (Figures 1 and 2) reveals several important points. First, all HIV-prevention interventions reviewed here are highly cost effective; that is, the cost per DALY is far less than one GDP per capita; and most interventions in Africa cost less than 30% of one GDP per capita and 40% in other regions. Second, all HIV-prevention interventions reviewed are cost effective when compared to other life saving interventions including HIV treatment, which is consistent with other recent comparative results [43-45]. Third, a "handle with care" caveat is absolutely essential. Based on the literature results, as they are published, we cannot differentiate with certainty which intervention types of the three (behavioral, biomedical and structural) seem to be more CE than others, all the more so because the studies reviewed did not consider interactions between different types of interventions. Likewise, inter-country or inter-study comparisons are problematic in the literature because the base-case scenarios are different, and there are many uncertainties and unknowns when deciding how to model the CE of HIV prevention. Most importantly, the question of the actual effectiveness of many of the modeling exercises may be misleading for policymakers. In the remainder of this section, we point out some of the specific limitations, first by type of intervention, and then by specific issue.

### Behavior change interventions

Because only modest evidence of effectiveness exists for school-based interventions in terms of HIV prevention [46-48], as well as limited costing data [48], it is not surprising that we found only one generalized CE study focusing n school interventions [21]. International experience with HIV prevention programs at the school level is ample, but it is a controversial subject. Comprehensive literature reviews for developing countries [46,47,49,50] suggest that programs at the school level can have a positive impact on attitudes and knowledge of HIV/AIDS and other STIs. The results indicate that many of the interventions have not been fully successful in obtaining the stated goals of modifying the risk behaviors and postponing initiation of sexual activity, reducing the number of sexual partners, and reducing the number of unplanned adolescent pregnancies. In addition, many programs have not strongly demonstrated an effect on consistent condom use or sexual abstinence. Hence, with such limited effectiveness information, it is difficult to show realistic CE results.

The evidence for the effectiveness of voluntary counseling and testing on HIV incidence is also ambivalent. Although some experimental evidence has shown that VCT can promote preventive behaviors [51], a recent meta-analysis of VCT in developing countries concludes that there is only a moderate effect of VCT in reducing unprotected sex and inconclusive evidence for the effect of VCT on the number of sexual partners [52]. Prior systematic reviews and meta-analyses showed similar mixed results [53]. A cluster-randomized trial in Zimbabwe showed that uptake of VCT services continues to be low, prompting consideration of new strategies to improve uptake, such as workplace-based alternatives [54]. Nevertheless, we found a study in Colombia [55] that presents a decision tree to compare CE of three strategies for HIV screening of pregnant women: voluntary, universal and optional. The study includes all women with unknown HIV status admitted for child birth in the country. For every HIV-positive newborn case detected, the universal screening strategy is less costly (US$ 17) than the optional (US$ 24) and the voluntary (US$ 38) strategies. However, as this study does not present costs per HIA it was not presented in the results section.

### Biomedical interventions

It is not unexpected that no studies of CE on microbicides have been published in the last four years, given the mostly disappointing news from the clinical trials [56-58]. Similarly, progress on a vaccine for HIV has been slow [59]. An international vaccine trial was discontinued in its second phase after interim analysis showed that the vaccine efficacy could not be obtained [60,61] and that it may actually make the subjects more susceptible to HIV [62,63]. So far, the only vaccine candidate to complete a phase III efficacy trial had no protective effect [64]. Furthermore, experimental and policy challenges to the development of a vaccine [65] make the hope more uncertain [63]. Nevertheless, vaccine research has seen massive investment estimated at about US$ 800 million a year [66], as well as increased coordination efforts [67]. Modeling estimates suggest that even a modestly efficacious vaccine could have a substantial impact on controlling the epidemic and substantial financial savings; thus, the reason to continue investing resources on developing an effective vaccine [68].

Furthermore, other analysis on vaccines by Berndt and colleagues [69] highlight the key role played by product pricing, uptake rates, and efficacy in the CE analysis process. In particular, they emphasize that CE estimates are sensitive to market prices; but, in turn, market prices are sensitive to volume discounts which can be achieved with more cost effective interventions. The authors also find that duration of protection would have a strong effect on the CE of a vaccine.

Only one study [24] explicitly modeled the interaction between ART and prevention, although some effectiveness evidence does exist [70-73]. ART reduces viral load, but it also increases physical wellbeing, and patients' ability to engage in sexual activity. Hence, the interactions between providing ART and continuing prevention efforts should be further explored in terms of CE.

STIs such as herpes simplex virus type 2 (HSV-2) may increase HIV transmission [74]. The earliest study conducted in Mwanza, Tanzania suggested that when STIs are treated, HIV infection declined by almost 40% over a two year period [75]. Following this result, STI treatment was included in the catalogue of HIV prevention measures endorsed by the WHO and UNAIDS [76]. However, another RCT in Rakai Uganda showed contradictory results [77] and other studies have not replicated the Mwanza level of efficacy [78-81]. Prevention of STIs follows in general the same recommendations as HIV prevention: reduction of number of sexual partners, condom use, etc.; thus, prevention of STIs could in principle also help to prevent HIV infection [82].

While some of the studies reviewed here suggest that treating STIs can be cost-effective, the epidemiological debate continues about the effectiveness of treating STIs as a way to prevent incident cases of HIV. The controversy, over whether treating STIs has any impact on HIV, casts doubt on the CE results reviewed here, or at least provides further sources of uncertainty around them. Until the issue is settled, one alternative for researchers may be simply to limit the analysis to costs per STI treated. STI prevention and treatment interventions can be beneficial and cost effective in their own right. For example, an intervention in Managua, Nicaragua [83] provided vouchers to sex workers, their regular clients and/or partners, transvestites, and male glue-sniffers. It increased the treatment rate of STIs (gonorrhea, Chlamydia, syphilis, and trichomoniasis) from 15% to 92% by making the services more affordable and of higher quality. Moreover, the study showed that the ICER was US$ 103 per STI cured. If personnel costs were reduced by 50%, the incremental CE ratio would fall to US$ 83 per STI cured [83]. This study was not analyzed in the results section as it did not provide costs per HIA or costs per DALY averted.

Among the biomedical interventions, male circumcision stands out as highly cost-effective for the level of efficacy demonstrated in the three RCTs. The five CE studies [33-37] conducted in high-level generalized epidemics show that investment in this intervention can result in averting significantly large numbers of new HIV infections. However, for concentrated epidemics, studies are needed on the effectiveness of MC among men who have sex with men and on the CE among infants and among heterosexual adults.

### Structural interventions

Structural interventions include policy tools such as changing the tax structure, sex industry regulation, property rights and access to credit for women. Many of these programs have been evaluated recently, but they have not directly addressed HIV prevention, even though they may have had the potential to do so. For example, Cohen and Dupas [84] present results from Kenya where in-kind incentives and price subsidies were used for malaria prevention. Women were randomized to free or three (intervention) price-levels of insecticide-treated bed nets if they attended a health post where general health and HIV prevention information was provided. They showed that higher incentives (lower prices) directly increased the uptake of bed nets. However, they did not directly link attendance to treatment and PMTCT in the form of nevirapine for HIV-positive mothers.

Similarly, experimental research in Malawi by Thornton [85] suggests that even a small economic incentive (as little as one tenth of daily wage) can increase the percentage of people returning for their HIV test results to about 80% (compared to the 39% of participants who return for HIV test results without an incentive). Furthermore, HIV-positive individuals who learn their infection status are three times more likely to purchase condoms. However, the number of condoms purchased was small, only two on average. Testing alone does not seem to be as cost effective as other interventions [85].

Although few structural interventions measure HIV infections averted (HIA), the main outcome of interest, structural interventions in general and conditional cash transfers [86] in particular could have a great potential to alter behaviors and prevent HIV. Randomized controlled trials are needed to test various hypotheses regarding the optimal level of incentives, the best implementation strategies, and the best conditions for testing and treatment services. Structural and environmental interventions seem to have the potential to improve the effectiveness and CE of currently proven and new interventions. More experimental and modeling evidence is needed to further explore the effects of changes in laws, taxes, and economic incentives on HIV prevention. A promising avenue may be to add HIV and/or STI components to conditional cash transfer interventions that have already proved to be effective in changing health behaviors in low- and middle-income countries. Prospective trials need to be designed carefully, measuring potential benefits as well as the possibility of unintended harm, e.g. spending the extra cash to buy drugs or alcohol. Also, a great challenge in conditional cash transfer (CCT) programs is the supply side (quality of services); this is particularly important for populations such as MSM who often do not use public services because of the discrimination they encounter.

### Intervention bundles and synergies

Interventions in the context of programs are usually bundled; for example VCT and condom promotion may be provided at the same time. This not only makes it more difficult to evaluate the impact of a single intervention, it suggests that effectiveness is dependent not only on how an intervention is implemented, but also on what other interventions are bundled with it [3,87]. Better evaluation designs (including multiple-arm controlled trials) may be required so that each component, their synergies, as well as total effects, can be estimated. Only when information is available about the likely synergies between different prevention interventions will CEA realize its potential in helping to identify the optimal package or the "right mix" for a given country or situation.

The synergies between prevention and treatment can also be analyzed using a CE approach. At a basic level, it is clear that if prevention is enhanced, there will be less treatment needed in the future. As treatment efforts become more widespread, prevention among HIV positive individuals is essential to reduce HIV transmission. As noted already, we did not find any CE studies on prevention for or with positives. The typology needs to be updated also: can prevention for people with HIV be similar, or are there prevention interventions that are specific and different for the positive population? In general, we have little empirical information about the interactions between different prevention and treatment strategies [88]. More research is needed in the area of prevention for positives.

### Scale, cost functions and nonlinearities

Many unknowns remain in the relationship between scale and costs [89]. Particularly, in high-level generalized epidemic settings, there is still no clear CE evidence on how to achieve a scale sufficiently large to have a major impact on HIV incidence. For example, male condoms continue to be one of the most widely used interventions, yet there is no recent CE evidence on how to achieve higher rates of condom utilization.

Unit costs can decrease considerably with scale across many programs in different settings, but there can be important differences within the same country [90]. Also, non-linear relationships between unit costs and scale can occur [91]. The shape of the cost function [92] can reveal very different unit costs, and thus very different CE, at different scales of implementation. These effects are almost universally ignored in the studies reviewed here and for the DCPP [3,89]. Non-linear effectiveness with scale is also an under-explored area, touched upon just by some of the MC studies reviewed here. Modeling work by Martina Morris and others [93] suggests that the relationship between behavior change and epidemic behavior can be far less linear than typically assumed in most CE models.

For some basic interventions (such as male condoms), there are estimates of the threshold at which the reproductive rate (R_0_) for the epidemic (i.e., the number of secondary cases caused by a primary case over the lifetime of that infected person) goes below unity [94]. R_0 _may be useful as a theoretical outcome measure [95]. However, a number of model assumptions are needed to estimate at which point an intervention reaches R_0 _< 1. To estimate it empirically would require precise knowledge of how HIV is transmitted in each setting, and detailed information on sexual behaviors and networks. Moreover, the relevant question is whether a specific package of prevention interventions can achieve an R_0 _< 1; it is a less useful concept for one intervention considered in isolation.

The specific effects of condom use on the dynamics of the epidemic are likely to be nonlinear. Similarly, the effects of ART on HIV transmission, due to its impact on longevity and sexual activity, are another example of nonlinear impacts which have been overlooked.

### Lack of standard, transparent methods

The literature continues to have only a few studies per type of intervention, not always with standardized and transparent methods, which does not facilitate comparison of results. Thus, the limited CE evidence is difficult to use in policy planning. Few studies report how their results have been used in weighing policy alternatives. Moreover, the studies do not generally discuss how to interpret results in terms of thresholds, nor do they explore budgetary constraints and acceptability curves that would provide useful information to policymakers.

Modeling techniques to assess the CE of one or more interventions continue to be widely used. Models are a useful resource to estimate final outcomes when data are not available. However, modeling on untested assumptions can be a disservice for policy because it may raise false expectations of programs, as there are still many empty cells in the effectiveness matrices [96]; thus, many CEA studies can be highly speculative. More efforts should be made to compare models and develop international guidelines to provide readers with tools to evaluate the results and assumptions of a model. The WHO-CHOICE project [42] has provided some guidelines. Additional challenges are to have the guidelines followed, and finding the right tools to translate results from complex models to decision-makers.

### Publication bias

Effectiveness studies that fail to show effectiveness would have no reason to include a CE component because an intervention without demonstrable effectiveness would have an infinite cost per unit of benefit. Thus, some of the peer-based programs, school education studies, STI treatment or the comprehensive community-prevention trials that have failed to show benefit should be assigned an infinity cost per DALY estimate [3]. Nevertheless, some CEA studies continue to include an implicit cost per unit of benefit based on selective assumptions.

## Conclusion

A number of CE studies of HIV-prevention interventions have become available in the past four years. As a body of work, they provide important information by analyzing CE ratios which depend upon a variety of factors including epidemiological characteristics, population targeted, coverage, unit prices and the technical efficiency for implementation. Focusing on the interventions that are most likely to be cost effective could help countries move from small, isolated programs to large-scale, comprehensive prevention efforts. Given the limited resources, as new and potential interventions are shown to be effective, they need to be fully analyzed in terms of their CE. This review shows that many HIV-prevention interventions are cost effective in absolute terms (using costs per DALY averted), and also in country-specific relative terms (measured as percentage of GDP per capita).

Nevertheless, a number of observations can be made about the state of the literature. First, we do not have CE studies for each epidemic type, even for the most commonly recommended or implemented HIV-prevention interventions. Second, scale-up scenarios often assume that relationships are linear, when there may be nonlinearities and economies and/or diseconomies of scale. Third, substantial uncertainty of unit costs and effects result in uncertain CE estimates, which makes difficult comparisons among interventions (even with the aid of league tables); we need to recognize that the degree of error (both measurement and estimation) can be substantial. Fourth, CEA in HIV prevention lacks consistency among studies. The international efforts on CE have developed useful guidelines, but few researchers use them. Fifth, CEA is useful as it presents results in a way that shows potential impact given a budget to spend on mutually exclusive alternatives. The problem is that in the real world the alternatives are seldom mutually exclusive due to special interests, political and equity concerns. Decision-making at the local level is never simply choosing the most cost effective alternative.

More than 25 years into the AIDS epidemic and billions of dollars of spending later, there is still much work to be done both on costs and effectiveness to adequately inform HIV prevention planning.

## List of abbreviations used

AIDS: (Acquired Immune Deficiency Syndrome); ART: (Antiretroviral Therapy); AZT: (Azidothymidine (zidovudine)); BF: (Breast Feeding); CE: (Cost-Effectiveness); CEA: (Cost-Effectiveness Analysis); CCT: (Conditional Cash Transfers); CSM: (Condom Social Marketing); CSW: (Commercial Sex Workers); DALY: (Disability-Adjusted Life Year); DCPP: (Disease Control Priorities (in Developing Countries) Project); FC2: ((Nitrile) Female Condom); FSW: (Female Sex Worker); GDP: (Gross Domestic Product); GUD: (Genital Ulcer Disease); HIV: (Human Immunodeficiency Virus); HIA: (HIV Infection Averted); HSV-2: (Herpes Simplex Virus type 2); ICER: (Incremental Cost-Effectiveness Ratio); IDU: (Injecting Drug User); IEC: (Information, Education and Communication); MSM: (Men who have Sex with Men); MSW: (Male Sex Worker); MC: (Male Circumcision); MTCT: (Mother-To-Child Transmission); NVP: (Nevirapine); PEPFAR: (The (United States') President's Emergency Plan for AIDS Relief); PMTCT: (Prevention of Mother-To-Child Transmission); PPT: (Presumptive Periodic Treatment); QALY: (Quality-Adjusted Life Year); RCT: (Randomized Controlled Trial); SSA: (Sub-Saharan Africa); STD: (Sexually Transmitted Disease); STI: (Sexually Transmitted Infection); TB: (Tuberculosis); UNAIDS: ((Joint) United Nations Program on HIV/AIDS); VCT: (Voluntary Counseling and Testing); WHO: (World Health Organization).

## Competing interests

The authors declare that they have no competing interests.

## Authors' contributions

All the authors designed the search strategy, and contributed to identifying eligible studies. OG and AC conducted data extraction based on a checklist for cost-effectiveness studies. OG wrote the first draft narrative analysis by theme. RW wrote substantive earlier drafts of the effectiveness components. SB reviewed and made contributions to the final draft. All authors reviewed and approved the final version of the paper.

## Supplementary Material

Additional file 1 Click here for file
