# Supplementary material for: HIV prevention cost-effectiveness: a systematic review
Source: BMC Public Health. 2009 Nov 18;9(Suppl 1):S5. doi: 10.1186/1471-2458-9-S1-S5 (PMC2779507; doi:10.1186/1471-2458-9-S1-S5)
Supplement: Additional file 1 [file 1471-2458-9-S1-S5-S1.doc]

# Cost-effectiveness of HIV-prevention interventions, 2005-2008

| ***Citation/setting*** | ***Intervention type*** | ***Target population / issue addressed*** | ***Epidemic profile*** | ***Main results*** |
| --- | --- | --- | --- | --- |
| **BEHAVIOR CHANGE INTERVENTIONS** | | | | |
| Hausler, Sinanovic et al. 2006; Cape Town, South Africa [20]. | VCT | Self-presenting and antenatal clients as well as TB and STI patients; retrospective annual cost data in 3 care facilities. | Generalized high-level | ProTEST intervention CE range: US$ 67 per HIV infection averted (HIA) in the STI clinic to US$ 112 in the community health clinic. |
| Hogan, Baltussen et al. 2005; sub-Saharan Africa & South East Asia [21]. | VCT, School-based education, etc. | Youth aged 10-18 years. Regular sessions to all students. Costs include teacher training. | Generalized high-level & Concentrated | US$ 6704 per HIA or US$ 376 per DALY (assumes 95% coverage). |
| John, Farquhar et al. 2008; Nairobi, Kenya [22] | VCT | Couple counseling for PMTCT; data on time and costs of pregnant women and couple counseling. | Generalized low-level | Infant HIA for US$ 483 using any of the two VCT options explored in the study: individual vs. couple counseling. |
| Vickerman, Kumaranayake et al. 2006; Odessa, Ukraine [23]. | Treatment of addictions | Injecting drug users (IDUs). Economic costs by coverage and IDU HIV prevalence rate. | Concentrated | 792 HIA at US$ 97 per HIV infection (at 20-38% service coverage and IDU HIV prevalence of 54%). |
| **BIOMEDICAL INTERVENTIONS** | | | | |
| Over, Marseille et al. 2006; India [24]. | ARTs | Modeling of 3 ART policy options to predict course of the epidemic in the absence of expanded ART availability: improved adherence to therapy; treatment for PMTCT including husbands; and subsidies for ART for people living below the poverty line. | Concentrated | Cost per life year saved compared with baseline scenario is US$ 146 for ADHERE, US$ 199 for MTCT+, US$ 286 for below poverty line (BPL). |
| Reynolds, Janowitz et al. 2006; Sub-Saharan Africa [25]. | PMTCT | Contraception as a strategy to prevent perinatal HIV transmission through decreasing unintended pregnancies which prevents HIV-positive births. Health system perspective with hypothetical sub-population during 1 year. | Generalized high-level | US$ 663 per HIV-positive birth averted (for the family planning strategy) vs. US$ 857 per HIV-positive birth averted (with single-dose nevirapine regime). |
| Soorapanth, Sansom et al. 2006; South Africa [26]. | PMTCT | Cohort of 100,000 women tested at 28, 34 and 36 weeks of pregnancy with different combinations of prenatal prophylactic and pediatric ART regimens. | Generalized high-level | HIV rescreening would prevent additional infant infections and result in net savings when zidovudine plus single-dose nevirapine or single-dose nevirapine is used for perinatal HIV prevention, and ART was available to treat perinatally HIV-infected children. |
| Maclean and Stringer 2005; Sub-Saharan Africa [27]. | PMTCT | Hypothetical cohort of 40,000 pregnant women. BF for 6 months with daily infant nevirapine (NVP) prophylaxis; maternal combination ART during pregnancy and for 6 months of BF; and maternal combination ART only for women who meet CD4 criteria. Each was compared to: BF for 12 months; BF for 6 months; and formula feeding for 12 months. | Generalized high-level | Providing daily infant NVP cost an additional 93,638 dollars and generated 1183 additional QALYs: ICER=US$ 79/QALY  Maternal combination ART was potentially very effective but too costly for most resource-poor settings (ICER: US$ 87/QALY). |
| Teerawattananon, Vos et al. 2005; Thailand [28]. | PMTCT | Modeling of hypothetical cohort of 100,000 pregnancies. Decision model assessed CE of 4 ART regimens given in addition to VCT for PMTCT: a) Zidovudine (AZT); b) Nevirapine (NVP); c) a combination of AZT for early antenatal attenders and NVP for late arrivals; and d) combined administration of AZT and NVP and to assess the incremental CE of adding a second VCT session in late pregnancy. | Generalized low-level | One VCT session with AZT+NVP averts 337 cases of infection at US$ 556 per case averted, while two VCT with the same drug regimen averts 16 additional cases at cost of US$ 1266 per infection averted. The incremental CE ratio of moving from 1VCT, AZT+NVP to 2VCT, AZT+NVP is US$ 16,000 per additional averted case, which is much lower than the recommended threshold value for HIV infection averted in Thailand. |
| Vickerman, Terris-Prestholt et al. 2006; South Africa, Johannesburg [29]. | Treatment of STIs | Female sex workers (FSW); mathematical model, fitted to epidemiological data, to estimate HIV & STI cases prevented. CE of intervention with and without periodic presumptive treatment (PPT). | Generalized high-level | US$ 2093 per HIA or US$ 78 per DALY; US$ 85 per DALY if FSW treated w/ syndromic management; incremental cost of adding PPT was US$ 31 per DALY. |
| Price, Stewart et al. 2006; Malawi, Lilongwe [30]. | Treatment of STIs | Male clinic attendees with urethritis and genital ulcer disease (GUD) were randomized to receive treatment for trichomoniasis or placebo in addition to the standard of care. | Generalized high-level | Expanding STI services to include trichomoniasis represents an excess cost of US$ 350.2 and prevents 22.7 cases of HIV in comparison to the status quo; this is equivalent to an ICER of US$ 15.43. |
| Oster 2005; Africa [31]. | Treatment of STIs | Modeling based on decreasing transmission  rates, involves treating other untreated (bacterial) sexually transmitted infections. Intervention modeled on the Mwanza, Tanzania [75]. | Generalized | Intervention would save 291 million life years with 13 million HIA. Achieved at a cost of US$ 3.67 per life year, and around US $ 78 per infection. Sexual behavior intervention slightly less effective, preventing 6 million infections at a cost of US$ 16.82 per life year and US$ 436 per HIA. |
| White, Orroth et al. 2008; East and West Africa [32]. | Treatment of STIs | STI simulation model fitted to four HIV epidemics to estimate population-attributable fractions of incident HIV attributable to STIs. Cost per HIA compared with lifetime HIV treatment costs (US $3500). | Generalized  low- and high-level | Cost per HIA range: US$ 321-1665. Curable STI interventions remain cost-saving when compared to lifetime HIV treatment costs in generalized HIV epidemics, populations with high-risk behaviors & low male circumcision rates. |
| Kahn, Marseille et al. 2006; South Africa, Gauteng Province [33]. | Male circumcision (MC) | General adult male population. 1000 newly circumcised adult men followed dynamically over 20 years to estimate HIV incidence reduction. | Generalized high-level | US$181 per HIA (at 25.6% adult HIV prevalence).  US$ 551 per HIA (at 8.4% adult prevalence). |
| Gray, Li et al 2007; Uganda, Rakai [34]. | MC | MC for males with impact on HIV incidence reduction for both males and females aged 15+. Efficacy (40-60%) and coverage rates (25-100%) varied. | Generalized high-level | 19-58 surgeries per infection averted over 10 years; US$ 1269-3911 per HIA based on (40-60%) efficacy of circumcision and 75% service coverage. |
| Martin, Bollinger et al. 2007ab; Lesotho & Swaziland [35, 37] | MC | Adult population. Country-wide CE of scaling up MC among males (ages 15 49) to 57.5 percent coverage (i.e., to reduce number of uncircumcised men by half) between 2008 and 2020. | Generalized high-level | US$ 292 per HIA in Lesotho; one HIV infection would be averted for every 6.1 MCs. US$176 per HIA in Swaziland; one HIV infection would be averted for every 4.1 circumcisions. |
| White, Glynn et al. 2008; sub-Saharan Africa [36]. | MC | Individual-based model fitted to the characteristics of illustrative high-HIV-prevalence population in sub-Saharan Africa The CE was calculated over 2-50 years. Future costs and effects discounted and compared with present value of lifetime HIV treatment costs (US$ 4043). | Generalized high-level | The cost per HIA by the default intervention targeted at 15–49 year olds, over 5, 10, 20, 30, 40 and 50 years, was US$ 974 (691–1964), 431 (308–842), 195  (143–356), 132 (100–232), 104 (81–179), and 89 (71–150) respectively. |
| Dowdy, Sweat et al. 2006; Brazil & South Africa [38]. | Female condom | Country-wide distribution of nitrile female condoms (FC2). Estimation of costs and impact of a female condom at different volumes of distribution. | Generalized high-level / concentrated | Brazil: US$ 20,683 per HIA.  South Africa: US$ 985 per HIA or US$ 18 per DALY. |
| **STRUCTURAL / ENVIRONMENTAL INTERVENTIONS** | | | | |
| Sweat, Kerrigan et al. 2006; Dominican Republic; Santo Domingo and Puerto Plata [39]. | 100% Condom  plus  communication campaign  plus  law changes | CSWs and clients in 41 sex establishments. One intervention includes: community mobilization, promotional media and interpersonal communication and counseling. A second intervention adds enhanced STI clinical services, monitoring and evaluation system with graduated sanctions on sex establishment’s owners. | Generalized low-level / concentrated | US$ 10,856 per HIA and US$ 457 per DALY in Puerto Plata (structural approach with law changes); versus US$ 28,208 per HIA and US$ 1,186 per DALY saved in Santo Domingo (traditional IEC) |
| Fung, Guinness et al. 2007; India, Ahmedabad [40]. | Empowerment / Social / Peer-based programs | CSW strategies with peer educators: increase knowledge of HIV/AIDS and STIs, improve STI treatment of CSW and clients, increase safer practices, environment improvement. Compared with no intervention. | Low-level | Cost per HIA is US$ 59.3 (33.7-133.4) (peer educator valued as financial cost) and US$ 97.7 (55.6-128.5) (peer educator valued as economic cost). US$ 3.1 (1.9-7.5) to 5.5 (3.1-12.3) per DALY. |
| Hogan, Baltussen et al. 2005; Sub-Saharan Africa [21]. | Mass media | General population. TV and radio episodes and inserts in key newspapers, repeated every two years. Effectiveness: proportion of population exposed to campaign. | Generalized high-level | Mass media US$ 58 per HIA or US$ 3 per DALY (ICER compared to no intervention). |

**Notes**: The table presents a complete summary of the CE studies found and reports costs per HIV infection averted, and costs per DALYs averted. Hogan, Baltussen et al (2005) [21] assessed CE of several interventions discussed in this review. They distinguish between treating STIs in the general population and combining peer education with treatment of STIs among sex workers. They used the uptake of condoms, among others, as a measure of the impact of preventive interventions rather than using condoms as an intervention. As such, that study was not considered as a CE study in the 100% condom category. ART=antiretroviral treatment, BF=breast feeding, CE = cost-effectiveness CSW=commercial sex workers; DALY=disability-adjusted life year; HIA=HIV infection averted; IEC=information, education & communication; ICER= incremental cost-effectiveness ratio; IDUs=injecting drug users; SSA=sub-Saharan Africa; STI=sexually transmitted infection; TB=tuberculosis; QALY=quality-adjusted life year. If the target population is not specified, then it is the general population.

**Epidemic profiles:** low-level epidemic (adult HIV prevalence < 1% and highest prevalence in key population<5%); concentrated epidemic (adult HIV prevalence < 1% and highest prevalence in key population>5%); generalized low-level epidemic (adult HIV prevalence < 10% and highest prevalence in key population<5%); and generalized high-level (adult HIV prevalence >=10% and highest prevalence in key population>5%)
